# NanoLAS: a comprehensive nanobody database with data integration, consolidation and application

**DOI:** 10.1093/database/baae003

**Published:** 2024-01-31

**Authors:** Shuchang Xiong, Zhengwen Liu, Xin Yi, Kai Liu, Bingding Huang, Xin Wang

**Affiliations:** College of Big Data and Internet, Shenzhen Technology University, Shenzhen 518118, China; College of Big Data and Internet, Shenzhen Technology University, Shenzhen 518118, China; College of Big Data and Internet, Shenzhen Technology University, Shenzhen 518118, China; College of Big Data and Internet, Shenzhen Technology University, Shenzhen 518118, China; College of Big Data and Internet, Shenzhen Technology University, Shenzhen 518118, China

## Abstract

Nanobodies, a unique subclass of antibodies first discovered in camelid animals, are composed solely of a single heavy chain’s variable region. Their significantly reduced molecular weight, in comparison to conventional antibodies, confers numerous advantages in the treatment of various diseases. As research and applications involving nanobodies expand, the quantity of identified nanobodies is also rapidly growing. However, the existing antibody databases are deficient in type and coverage, failing to satisfy the comprehensive needs of researchers and thus impeding progress in nanobody research.

In response to this, we have amalgamated data from multiple sources to successfully assemble a new and comprehensive nanobody database. This database has currently included the latest nanobody data and provides researchers with an excellent search and data display interface, thus facilitating the progression of nanobody research and their application in disease treatment.

In summary, the newly constructed Nanobody Library and Archive System may significantly enhance the retrieval efficiency and application potential of nanobodies. We envision that Nanobody Library and Archive System will serve as an accessible, robust and efficient tool for nanobody research and development, propelling advancements in the field of biomedicine.

**Database URL**: https://www.nanolas.cloud

## Introduction

Nanobodies, a unique subclass of antibodies discovered in camelid animals (1), are composed solely of a single heavy chain’s variable region, contributing to their compact structure and significant therapeutic advantages. The diverse properties of nanobodies underpin their broad application potential in biological research and disease treatment (2, 3). Nanobodies exhibit high specificity, solubility, stability and antigen affinity with low toxicity and immunogenicity (4). Nanobodies can maintain structural stability in acid, alkali, heat, salt and other environments, which makes nanobodies have good stability in both internal and external environments and can improve their application in the biomedical field. Nanobodies can be completely dissolved in water, physiological saline and other solutions. This makes the preparation and application of nanobodies more convenient (5). More importantly, nanobodies can bind to antigens to form high-affinity complexes. This allows nanobodies to effectively recognize and bind target antigens and exert their therapeutic and diagnostic effects (6). Furthermore, the small size allows nanobodies superior tissue penetration (6, 7), making them advantageous in disease treatment and molecular imaging (7–9). Nanobodies’ properties and smaller size enable effective tissue penetration, easy engineering, multimeric structure generation and application in diverse fields, including cancer treatment and Coronavirus Disease (COVID-19) drug development (6, 10–16).

Currently, a variety of nanobodies are already in clinical trials for the treatment and prevention of COVID-19. For example, bamlanivimab (LY-CoV555), a nanobody developed by AbCellera Biologics and the National Institute of Allergy and Infectious Diseases’ Vaccine Research Center, has been approved to treat COVID-19 (17). Another nanobody against COVID-19, 20G6, developed by the Institute of Microbiology, Chinese Academy of Sciences, showed broad-spectrum neutralizing activity against severe acute respiratory syndrome coronavirus 2 (SARS-CoV-2) in mouse models, including against Omicron mutant strains (18). Nanobodies are a potential new antiviral therapy (19). They are still in the early stages of development, but they have the potential to be an effective way to treat and prevent COVID-19. It is foreseeable that nanobodies will be the next innovation point in biomedicine. The establishment of a comprehensive and comprehensive nanobody database will make the research of nanobodies more convenient.

The continuous advancement in nanobody research and applications has led to a rapid accumulation of nanobody data over recent years. Current databases, such as Protein Data Bank (PDB), Integrated Nanobody Database for Immunoinformatics (INDI), the international ImMunoGeneTics information system for immunoglobulins or antibodies (IMGT) and the Single Domain Antibody Database (SdAb-DB), among others, house vast volumes of nanobody information. However, these databases may fall short in terms of data type and coverage.

Furthermore, the heterogeneity, inconsistency and lack of interoperability of data across different databases pose additional challenges for researchers. Each database follows its unique data format and structure, obliging researchers to invest substantial time and effort in data processing and integration when using multiple databases. Some databases do not even offer a user-friendly interface, complicating and prolonging the data query and analysis process.

To address these limitations, we propose the creation of a new nanobody database—Nanobody Library and Archive System (NanoLAS). This initiative aims to satisfy the scientific community’s need for a more comprehensive and in-depth understanding of nanobodies. NanoLAS will integrate and standardize nanobody data from diverse databases, offer a user-friendly, efficient and interactive query and analysis platform and facilitate the further development of nanobody research.

## Materials and Methods

### Data collection

**Figure 1.**
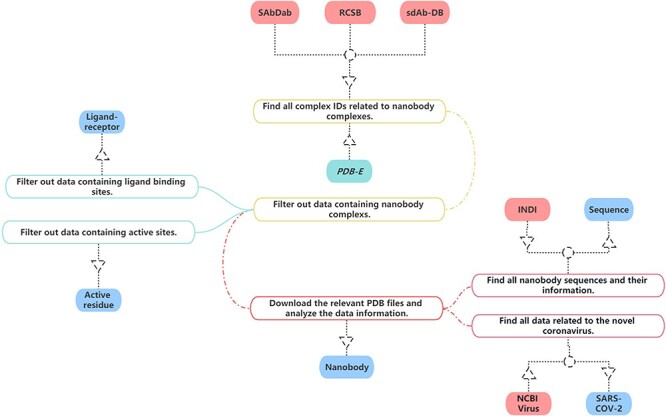
The process of data collection and processing of NanoLAS database.

In the construction of our nanobody database, we have sourced data from multiple publicly accessible bioinformatics databases in Figure 1. Given that each database employs unique information formats and content, it is necessary to carefully process and convert this information specifically to ensure uniformity.

For our project, we have gleaned all relevant nanobody protein structure information from the Research Collaboratory for Structural Bioinformatics (RCSB) PDB (20). Our selection and extraction process were based on a comprehensive set of screening criteria such as protein function, source, resolution and publication date.

Specifically, we first focus on common information about nanobodies, which is essential for studying nanobodies. This includes PDB Identification (PDB ID), total structure weight, atom count, modeled residue count, deposited residue count, molecule, method, sequence, source species and other information. Additionally, we obtain the structural files of nanobodies meanwhile preserving the information about the authors and their publication dates. Then, we focus on three key aspects of nanobody structures, ensuring that we gather thorough and comprehensive information. Firstly, concerning the ligand–receptor interactions in nanobodies, we extract relevant information, including the identification of ligands and receptors, binding sites, etc. This aids in a deeper understanding of the nanobodies’ functionality and activity in specific environments. Secondly, we focus on the active residues within nanobodies and crucial components for their functionality. We record the positions of active residues and the information about the sequences in which these residues are located. Lastly, we meticulously document the sequence information of nanobodies, encompassing amino acid sequences and the variable region amino acid sequences of nanobodies. This information is crucial for studying nanobodies and is key data for research such as nanobody screening and nanobody structure prediction models. This approach ensured that our collected data are representative and align with our research needs. Consequently, we successfully amassed a substantial amount of nanobody structure information, which forms a solid foundation for our database.

SdAb-DB (21) provides detailed and meticulously verified single-domain antibody sequences, sources, structures and associated biological information. We have selected and extracted nanobody-related data from SdAb-DB, comprising sequences, structures, affinities, sources and related biological information of single-domain antibodies.

Opig-SAbDab (Structural antibody database) (22) gathers detailed records of the source, sequence, 3D structure and other biological information of nanobodies. We extracted relevant nanobody data from Opig-SAbDab. These data include but are not limited to the amino acid sequence, structural model, source information and corresponding biological functions of nanobodies. Through the integration and utilization of these data, we have established a comprehensive and high-quality nanobody dataset in NanoLAS to support the research and application of nanobodies.

We extracted relevant SARS-CoV-2 data from the National Center for Biotechnology Information (NCBI) Virus database (23). Through the integration of this data, we enriched the SARS-CoV-2 entry in the NanoLAS database, providing a robust resource to support research and applications focusing on SARS-CoV-2.

Protein Data Bank in Europe (24) provides a variety of tools and services that allow users to search for and analyze structures available in the PDB archive. Some of its features include advanced search capabilities, analyses of macromolecular structures, sequence and structure alignment tools and links to other resources for further analysis. We utilize these tools and enriched the ligand–receptor and active residue entries in NanoLAS database.

Through the bulk download feature in INDI (25), we analyzed all nanobody sequences and integrated this data with the sequences gathered from RCSB, SAbDab and SdAb-DB. As a result, we have constructed a comprehensive nanobody sequence entry within our NanoLAS database.

### Website construction

**Figure 2.**
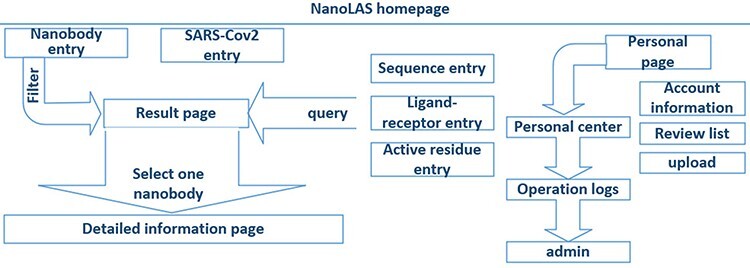
The architecture of NanoLAS website. iCAN website includes five search entries (left part) and a interface for users (right part).

In the construction of NanoLAS website (Figure 2), we utilized a modern technology stack for optimal efficiency and stability. The Vue.js framework, HyperText Markup Language, Cascading Style Sheet and JavaScript were employed for frontend development, creating user-friendly interfaces. Java Spring Boot was utilized for backend functions, while MySQL was selected for its powerful data processing capabilities. For molecular structure visualization, we used 3Dmol.js (26), a JavaScript library. The system operates based on the HyperText Transfer Protocol (HTTP) protocol, where user-initiated frontend requests are processed by the backend and the corresponding results are rendered on the frontend.

## Results

### Data overview

In the process of data integration, we have tried to include detailed information for each antibody sequence, such as source species, Complementarity-Determining Region (CDR) region lengths, etc.

For organism sources, the database contains nanobody data from wide variety of species, and *Lama glama* occupies the majority.

**Figure 3.**
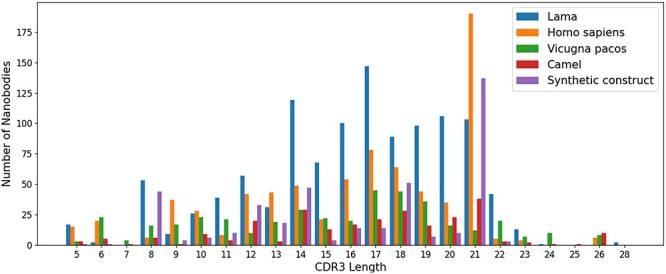
The distribution of CDR3 lengths in the NanoLAS database by source organism.

For sequences, we compared the length of CDR1–3 in NanoLAS database. While CDR1 and CDR2 do not show obvious length variations, CDR3 performs the most variable portion. CDR3 lengths span a wide range from 5 to 28 amino acids, with lengths 14, 16, 17 and 21 being the most represented in the database (Figure 3).

### Website features and operation interface

The NanoLAS database can be accessed at https://www.nanolas.cloud, and we also maintain a project repository on GitHub, facilitating developers and researchers for deep participation and contribution.

**Figure 4.**
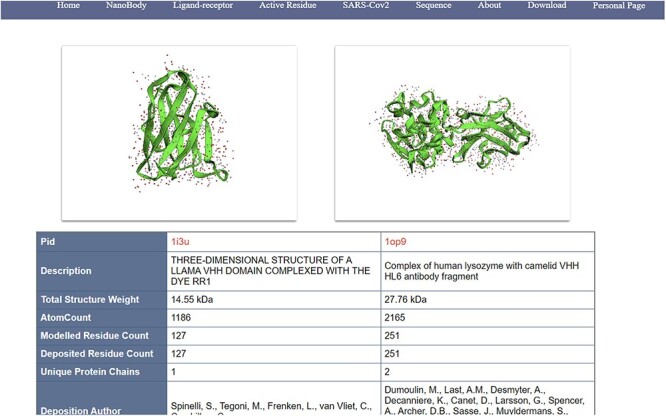
An example of information page of nanobodies (comparable/docking view).

(i) Search, filter and display of nanobodies (Figure 4): NanoLAS allows users to flexibly retrieve nanobody data in the database by providing various filters and search tools. Users can operate through four main entrances or the search box at the top. NanoLAS provides the following multiple retrieval methods for various nanobody sequences in the database:

Identifier of the nanobody source database, such as PDB ID.

Search by nanobody amino acid sequence.

Search by CDR region.

Year, source organism, etc.

Ligand receptor.

Active residues.

After retrieving the target antibody, users can choose different styles to observe the 3D structure, possibly using 3Dmol. The search results also include pid, total structure weight, total number of atoms, number of residues, description, publication date, etc. The search results also contain a download link for the pdb file, allowing users to download the pdb file for more detailed operations and analysis.

(ii) Comparison and analysis of nanobodies: NanoLAS provides users with the function of comparing and analyzing two different antibodies. If you need to compare two specific nanobodies, users can set the parameters in the search box. Through 3Dmol.js, we can intuitively compare the similarities and differences between different nanobodies in the 3D view and understand the structure of various nanobodies.

(iii) User data upload: We realize that there may be new nanobody data in the actual research and application process. Therefore, to comprehensively and meticulously collect nanobody data, NanoLAS specially provides a data submission page for nanobodies. On the nanobody data submission page, users can submit their nanobody information, such as Protein Data Bank Identification (PID), description, publication date, total number of atoms, etc. At the same time, to ensure the accuracy and reliability of the submitted data, we have set up a data review process. After users submit their nanobody information, we will conduct a data review. Only after the review, these data will be added to the database. We welcome and encourage all scholars and researchers to actively share their nanobody data, which will help promote the progress of nanobody research.

## Discussion

As a brand-new database specifically collecting nanobody information, NanoLAS has several advantages (Table 1). First, our database contains nanobody data from multiple sources, which is extensive and diverse, and can meet the search needs of different users. Although the structural data of nanobodies have been included in databases such as RCSB, IMGT/3Dstructure-DB and others, NanoLAS’s handling and presentation of data are more humanized and are easier for users to retrieve and understand. Secondly, NanoLAS provides a 3D view function, allowing users to intuitively view the spatial structure of nanobodies, which is very helpful for studying and understanding the structure and function of nanobodies. Furthermore, our comparative analysis function allows users to intuitively compare the differences between two or more nanobodies, assisting researchers in making scientific judgments. Finally, NanoLAS’s interface design is beautiful and easy to operate, providing users with a comfortable user experience.

**Table 1.** Comparison of NanoLAS and other databases

Database	Sequence search	Ligand–receptor search	Comparable/docking view	3D view	PDB files	Bulk download	Supports user sequence upload	Continuous update
NanoLAS	●	●	●	●	●	●	●	●
RCSB	●	○	○	●	●	●	●	●
NCBI	●	○	○	○	●	●	●	●
IMGT (3D structure DB)	○	●	○	●	●	●	○	●
INDI	●	●	○	○	●	●	○	○
sdAb-DB	●	●	○	○	●	●	●	○
Opig-SAbDab_Nano	○	●	○	●	●	●	○	●
Opig-COV(Coronavirus)	●	●	○	○	●	●	○	●

The table compares NanoLAS with other nanobody databases in terms of features about functionalities like search, upload and download. Each database is evaluated across multiple functions, highlighting NanoLAS’s features like search capabilities, user-friendly interface and extensive data coverage.

● Yes.

○ No.

With the progress of scientific research, nanobody sequence data are rapidly increasing and updating, it is important to note that the success of the database will depend on its continuous update and maintenance, as well as the addition of new features and tools based on user feedback and needs, and the NanoLAS database needs to take measures to regularly increase the collection and update to maintain the timeliness of the collected data.

## Conclusion

In this project, we successfully developed the nanobody database NanoLAS. It integrates nanobody data from multiple sources and provides a user-friendly interface, allowing users to easily query, analyze and visualize nanobody data, thereby improving research efficiency.

To continuously optimize and develop NanoLAS, we sincerely invite and look forward to various feedback and suggestions from users. We will strive to improve and expand NanoLAS to better serve the research and application field of nanobodies. For suggestions or queries, please reach out to us through our contact page.

## Data Availability

All data can be obtained at https://www.nanolas.cloud/.

## References

[1] Hamers-Casterman C. , Atarhouch T., Muyldermans S. et al. (1993) Naturally occurring antibodies devoid of light chains. *Nature*, 363, 446–448. 8502296 10.1038/363446a0

[2] Steeland S. , Vandenbroucke R.E. and Libert C. (2016) Nanobodies as therapeutics: big opportunities for small antibodies. *Drug Discov. Today*, 21, 1076–1113. 27080147 10.1016/j.drudis.2016.04.003

[3] Siontorou C.G. (2013) Nanobodies as novel agents for disease diagnosis and therapy. *Int. J. Nanomedicine*., 11, 4215–4227. 10.2147/IJN.S39428 PMC3818023 24204148

[4] Arbabi Ghahroudi M. , Desmyter A., Wyns L. et al. (1997) Selection and identification of single domain antibody fragments from camel heavy-chain antibodies. *FEBS Lett*., 414, 521–526. 9323027 10.1016/s0014-5793(97)01062-4

[5] Kolkman J.A. and Law D.A. (2010) Nanobodies—from llamas to therapeutic proteins. *Drug Discov. Today*, 7, e139–e146. 10.1016/j.ddtec.2010.03.002 24103724

[6] Kijanka M. , Dorresteijn B., Oliveira S. et al. (2015) Nanobody-based cancer therapy of solid tumors. *Nanomedicine (Lond)*, 10, 161–174. 25597775 10.2217/nnm.14.178

[7] Muyldermans S. (2013) Nanobodies: natural single-domain antibodies. *Annu. Rev. Biochem*., 82, 775–797. 23495938 10.1146/annurev-biochem-063011-092449

[8] De Meyer T. , Muyldermans S. and Depicker A. (2014) Nanobody-based products as research and diagnostic tools. *Trends Biotechnol*., 32, 263–270. 24698358 10.1016/j.tibtech.2014.03.001

[9] Beghein E. and Gettemans J. (2017) Nanobody technology: a versatile toolkit for microscopic imaging, protein–protein interaction analysis, and protein function exploration. *Front. Immunol*., 8, 771. 10.3389/fimmu.2017.00771 PMC5495861 28725224

[10] Chakravarty R. , Goel S. and Cai W. (2014) Nanobody: the “magic bullet” for molecular imaging. *Theranostics*, 4, 386–398. 24578722 10.7150/thno.8006 PMC3936291

[11] Dumoulin M. , Conrath K., Van Meirhaeghe A. et al. (2002) Single‐domain antibody fragments with high conformational stability. *Protein Sci*., 11, 500–515. 11847273 10.1110/ps.34602 PMC2373476

[12] D’Huyvetter M. , Aerts A., Xavier C. et al. (2012) Development of 177Lu-nanobodies for radioimmunotherapy of HER2-positive breast cancer: evaluation of different bifunctional chelators. *Contrast Media Mol. Imaging*, 7, 254–264. 22434639 10.1002/cmmi.491

[13] Hassanzadeh-Ghassabeh G. , Devoogdt N., De Pauw P. et al. (2013) Nanobodies and their potential applications. *Nanomedicine (Lond)*, 8, 1013–1026. 23730699 10.2217/nnm.13.86

[14] Van Audenhove I. and Gettemans J. (2016) Nanobodies as versatile tools to understand, diagnose, visualize and treat cancer. *EBioMedicine*, 8, 40–48. 27428417 10.1016/j.ebiom.2016.04.028 PMC4919472

[15] Bessalah S. , Jebahi S., Mejri N. et al. (2021) Perspective on therapeutic and diagnostic potential of camel nanobodies for coronavirus disease-19 (COVID-19). *3 Biotech*, 11, 1–4. 10.1007/s13205-021-02647-5 PMC7820838 33500874

[16] De Vlieger D. , Ballegeer M., Rossey I. et al. (2019) Single-domain antibodies and their formatting to combat viral infections. *Antibodies (Basel)*, 8, 1. 10.3390/antib8010001 PMC6640686 31544807

[17] Chen P. , Nirula A., Heller B. et al., BLAZE-1 Investigators. (2021) SARS-CoV-2 neutralizing antibody LY-CoV555 in outpatients with Covid-19. *N. Engl. J. Med*., 384, 229–237. 33113295 10.1056/NEJMoa2029849 PMC7646625

[18] Feng B. , Chen Z., Sun J. et al. (2022) A class of shark-derived single-domain antibodies can broadly neutralize SARS-related coronaviruses and the structural basis of neutralization and Omicron escape. *Small Methods*, 6, e2200387. 10.1002/smtd.202200387 PMC9347709 35583124

[19] Najmeddin A. , Shapourabadi M.B., Behdani M. et al. (2021) Nanobodies as powerful pulmonary targeted biotherapeutics against SARS-CoV-2, pharmaceutical point of view. *Biochim. Biophys. Acta, Gen. Subj*., 1865, 129974. 10.1016/j.bbagen.2021.129974 PMC8325376 34343644

[20] Rose P.W. , Beran B., Bi C. et al. (2010) The RCSB Protein Data Bank: redesigned web site and web services. *Nucleic Acids Res*., 39, D392–D401. 21036868 10.1093/nar/gkq1021 PMC3013649

[21] Wilton E.E. , Opyr M.P., Kailasam S. et al. (2018) sdAb-DB: the single domain antibody database. *ACS Synth. Biol*., 7, 2480–2484. 30441908 10.1021/acssynbio.8b00407

[22] Schneider C. , Raybould M.I. and Deane C.M. (2022) SAbDab in the age of biotherapeutics: updates including SAbDab-nano, the nanobody structure tracker. *Nucleic Acids Res*., 50, D1368–D1372. 34986602 10.1093/nar/gkab1050 PMC8728266

[23] National Library of Medicine (US), National Center for Biotechnology Information . (2004) NCBI Virus. Bethesda, MD. https://www.ncbi.nlm.nih.gov/labs/virus/vssi/#/ (14 January 2024, last date accessed)

[24] Velankar S. , Best C., Beuth B. et al. (2010) PDBe: Protein Data Bank in Europe. *Nucleic Acids Res*., 38, D308–D317. 19858099 10.1093/nar/gkp916 PMC2808887

[25] Deszyński P. , Młokosiewicz J., Volanakis A. et al. (2022) INDI—Integrated Nanobody Database for Immunoinformatics. *Nucleic Acids Res*., 50, D1273–D1281. 34747487 10.1093/nar/gkab1021 PMC8728276

[26] Rego N. and Koes D. (2015) 3Dmol.js: molecular visualization with WebGL. *Bioinformatics*, 31, 1322–1324. 25505090 10.1093/bioinformatics/btu829 PMC4393526

